# Mechanical Contact Characteristics of PC3 Human Prostate Cancer Cells on Complex-Shaped Silicon Micropillars

**DOI:** 10.3390/ma10080892

**Published:** 2017-08-02

**Authors:** Brandon B. Seo, Zeinab Jahed, Jennifer A. Coggan, Yeung Yeung Chau, Jacob L. Rogowski, Frank X. Gu, Weijia Wen, Mohammad R. K. Mofrad, Ting Yiu Tsui

**Affiliations:** 1 Department of Chemical Engineering, University of Waterloo, 200 University Avenue West, Waterloo, ON N2L 3G1, Canada; bsbseo@uwaterloo.ca (B.B.S.); jrogowsk@uwaterloo.ca (J.L.R.); frank.gu@uwaterloo.ca (F.X.G.); 2 Departments of Bioengineering and Mechanical Engineering, University of California Berkeley, 208A Stanley Hall, Berkeley, CA 94720, USA; zjahed@berkeley.edu (Z.J.); mofrad@berkeley.edu (M.R.K.M.); 3 Department of Chemistry, University of Waterloo, 200 University Avenue West, Waterloo, ON N2L 3G1, Canada; jcoggan@uwaterloo.ca; 4 Department of Physics, The Hong Kong University of Science and Technology, Clear Water Bay, Kowloon, Hong Kong, China; cyyab@connect.ust.hk (Y.Y.C.); phwen@ust.hk (W.W.)

**Keywords:** nanoindentation, contact mechanics, deformation, silicon, pillars, PC3 cells

## Abstract

In this study we investigated the contact characteristics of human prostate cancer cells (PC3) on silicon micropillar arrays with complex shapes by using high-resolution confocal fluorescence microscopy techniques. These arrays consist of micropillars that are of various cross-sectional geometries which produce different deformation profiles in adherent cells. Fluorescence micrographs reveal that some DAPI (4′,6-diamidino-2-phenylindole)-stained nuclei from cells attached to the pillars develop nanometer scale slits and contain low concentrations of DNA. The lengths of these slits, and their frequency of occurrence, were characterized for various cross-sectional geometries. These DNA-depleted features are only observed in locations below the pillar’s top surfaces. Results produced in this study indicate that surface topography can induce unique nanometer scale features in the PC3 cell.

## 1. Introduction

The recent development of vertically-aligned micro- and nano-pillar arrays for a range of biological applications, such as circulating tumor cell (CTC) capturing devices [1,2,3,4,5,6,7,8,9,10,11], biosensors [12,13,14,15,16], and neural microprobe implants [17,18,19,20,21], have helped to stimulate active research initiatives in understanding how cells interact with these micro- and nanometer-scale pillars. In addition to overall cell responses to these small features, several recent works were focused on the effects of nuclear deformation induced by such pillars. Pan et al. [22] cultured neonatal Sprague Dawley (SD) rat bone marrow stromal cells on poly (lactide-co-glycolide) (PLGA) square-shaped micropillars and observed pillar-induced shape changes of the nuclei. They reported that the nuclei were severely deformed and would conform to the contours of the PLGA surface features. Their results indicate that gravitational force is not the primary driving factor that causes the cells to deform. Instead, Pan et al. suggested that the deformation is due to cytoskeletal stresses. Furthermore, they revealed that cells with severely deformed nuclei continue to survive and maintain the ability to differentiate. Hanson et al. [4] evaluated the nuclear deformation of various cell types that showed different nuclear stiffness characteristics on nanopillars. They showed that both the cell and nuclear membranes conformed to the shape of the patterned structures. These cells survived even with severe pillar-induced nuclear deformations and continued to proliferate and migrate on the patterned surface. However, as expected, the amount of nuclear deformation depends on the stiffness of the nucleus, with more compliant organelles exhibiting greater strain. Davidson et al. [23] studied nuclear deformation behaviors of two cancerous osteosarcoma cell lines (SaOs-2 and MG-63) surrounding poly-l-lactic acid (PLLA) micropillars, and compared them with healthy human osteoprogenitor cells. Their results showed that the nuclei of cancerous osteosarcoma cells deformed more than those of healthy cells. These deformations were already visible only six hours after cell deposition. In addition, the severely deformed cancer cells continued to undergo mitosis and cell division. Their results suggested that the primary driving force behind nuclear deformation is the pressure generated by the actin cap above the nucleus which pushes the nucleus downwards onto the nanopillars. 

While these works produced valuable information on how cell and nuclear shape changed with various patterned surfaces, there have been few insights into how pillar cross-sectional geometries affect the nuclear deformation characteristics and their internal structure. Experiments conducted in previous works were focused on solid core structures with high orders of symmetry, such as rounded rectangular [23,24] or cylindrical pillars [25]. As a result, they lack sharp points or edge features that can produce high mechanical stresses, and do not represent certain complex shapes that are often seen in platforms designed for biological applications [4,9,14,16]. Furthermore, the pillar material commonly used in previous nucleus deformation studies [23,24] was PLLA which has an elastic modulus significantly lower than silicon (~4.1 GPa [26] vs. ~190 GPa [27]). This polymeric material is also weak and soft with a tensile strength ~163 times lower than silicon (~43 MPa [26] vs. ~7000 MPa [27]). Therefore, PLLA pillars with a given geometry may not induce as significant a mechanical stress on the adhered cells as their silicon counterpart due to localized Hertizan deformation or plastic yielding [28]. A good analogy is a stiff and strong diamond spherical tip producing larger mechanical contact stress when pressed on an elastic half-space in comparison to the contact stress of a PLLA tip with the same geometry under the same loading force. This difference exists because the compliant PLLA tip deforms readily at the contact point, thereby increasing the contact area and reducing the localized stress. In addition, the pressure applied by the cells may alter the polymeric pillar's geometries by bending [25,29], a possibility which brings uncertainty when interpreting cell deformation results. Jahed et al. recently demonstrated that a nanometer-scale *Staphylococcus aureus* bacterial cell network can produce sufficient forces to mechanically deform polydimethylsiloxane (PDMS) micropillars [29], and even high-strength nanocrystalline nickel nanopillars [30].

**Figure 1:**
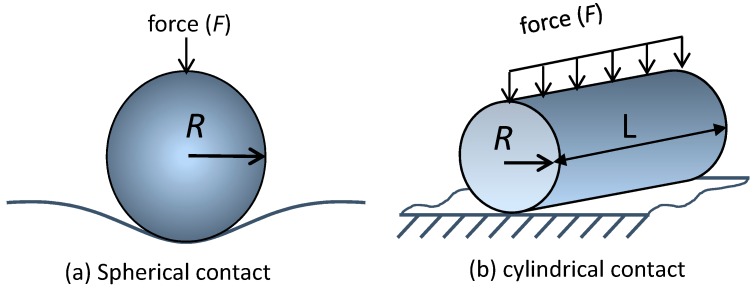
Schematic drawings of (**a**) spherical and (**b**) cylindrical tip contact geometries.

Determining the exact stress profiles within cells surrounding the pillars is difficult due to complex topographic features of the pillars, uncertainties in the magnitude/direction of external forces applied by the cytoskeletal elements on the adherent cells, the mechanical properties of live cells, and changes in cell contour as they spread around pillars. However, it may still be possible to appreciate the effect of pillar geometries on contact pressure by using simplified contact mechanic models as examples. Consider two Hertzian contact models for spherical and cylindrical tips on an elastic half-space [28] as schematically illustrated in Figure 1a,b, respectively. The maximum contact pressure (*p_max_*) produced by the external force (*F*) are given by the following equations:

(1) $$p_{max}\left(spherical\;tip\right)={\left(\frac{6FE^{*}{}^{2}}{\pi^{3}R_{s}{}^{2}}\right)}^{1/3}$$

(2) $$p_{max}\left(cylinder\right)={\left(\frac{FE^{*}}{\pi LR_{c}}\right)}^{1/2}$$

where parameters *R_s_* and *R_c_* correspond to the radius of the spherical and cylindrical tips, respectively. The length of the cylinder is represented by parameter *L*. The effective modulus (E*) is related to the elastic moduli (*E*) and the Poisson’s ratio (*v*) of the spherical/cylindrical tip material (*t*) and the elastic half-space (*h*) by the following equation:

(3) $$\frac{1}{E^{*}}=\frac{1-v_{t}^{2}}{E_{t}}+\frac{1-v_{h}^{2}}{E_{h}}$$

Equations (1) and (2) show that the tip radius maximum contact pressure relationship varies with R^−2/3^ and R^−1/2^, respectively. These models reveal that the maximum contact pressure (*p_max_*) increases as the spherical and cylindrical tip radii reduce. Hence, pillars with sharp corners or edges may induce significant contact pressure on the adherent cells.

The objective of this work is to gain an understanding of how prostate cancer (PC3) cell nuclei respond to sharp, mechanically stiff, and hard silicon surface structures with various cross-sectional profiles. Herein, complex C-shaped and hollow micropillars with different outer diameters were fabricated on hard silicon substrates. Each C-shaped pillar contains two sharp points and edges which may amplify the local mechanical stresses imposed on the cells by the cytoskeletal forces. This resembles the indentation contacts of sharp tips on viscoelastic materials. Silicon was chosen because of the fact that its elastic modulus, hardness, and yield strength are significantly higher than monolithic polymers, such as PLLA. This work is uniquely different from previous studies that used rectangular or cylindrical polymeric pillars: (1) silicon pillar cross-sectional geometries contain sharp and pointed features with tip radii in the nanometer-scale; (2) silicon pillars are ~45 times stiffer and their mechanical strength is ~160 times larger than polymeric pillars with the same geometry. This allows the mechanical stress to be concentrated on the cells without the uncertainty that the pillars will deform locally or bend when in contact with the cells. Prostate cancer cells were chosen because they are one of the most common cancers and leading causes of cancer death among men [31]. Each PC3 cell covers a large surface area in tens or hundreds of square microns when it is fully adherent. This allows a large number of silicon pillars to make contact with individual cells. DNA and cytoskeletal elements were subsequently stained and inspected with high-resolution confocal fluorescence microscopy. Results show that the DNA of PC3 cancer cells that have been incubated for at least 24 h would spread and cover the complex-shaped pillars. More importantly, DAPI (4′,6-diamidino-2-phenylindole)-stained nuclear micrographs reveal a new type of topography-induced feature at the nuclei locations. This feature appears as nanometer-scale slits emanating from the pillars, particularly near the sharp corners of the C-shaped pillars. To the best of our knowledge, this is the first time such small slits have been observed within human prostate cancer cells. Careful analysis showed that C-shaped pillars with smaller outer diameters resulted in a more frequent appearance of these slit features. A few slit structures were observed for hollow pillars that do not contain any sharp corners. Further inspection of the nuclei revealed that these slits were only observed at focal planes between the pillar top and the substrate surface. The lack of DAPI fluorescence signal from these slits suggests that they do not contain significant amounts of DNA. 

## 2. Materials and Methods

### 2.1. Substrate Preparation

Patterned silicon pillar arrays were prepared using standard microfabrication ultraviolet (UV) lithography and silicon etching techniques performed at the Hong Kong University of Science and Technology. The thickness of the AZ^®^7908 photoresist (AZ Electronic Materials, Merck KGaA, Darmstadt, Germany) was ~0.9 µm. After a soft-bake on a hotplate at 90 °C for 60 s, patterns were transferred to the substrate by UV light in an ASML Stepper 5000 (PHT-S1) (Veldhoven, The Netherlands) at an intensity of 500 mW for 0.66 s. The wafers were developed in FHD-5 (Fujifilm Electronic Materials Co. Ltd., Tokyo, Japan) for 60 s, followed by a hard-bake process at 120 °C for 30 min. Pillar patterns were transferred onto the silicon substrate using a Bosch anisotropic etch process [32] to render pillars of approximately 2.2 μm in height. Three different pillar geometries and dimensions were successfully manufactured. These include C-shaped pillars with outer diameters of approximately 1.2 and 2.7 μm, as well as hollow-shaped columnar structures with outer and inner diameters of approximately 5.6 μm and 3 μm. Detailed physical dimensions and spacing of these pillars are summarized in Table 1. These silicon substrates were rinsed with ethanol (70%) followed by phosphate-buffered saline (PBS) (Bio-Rad Laboratories, Mississauga, ON, Canada) prior to cell deposition.

### 2.2. Cell Culture and Staining

The human prostate cancer cell line PC3 was obtained from the American Type Culture Collection (ATCC, Manassas, VA, USA). Cells were passaged every three to five days upon reaching 80% confluence using 0.25% trypsin/0.05% ethylenediaminetetraacetic acid (EDTA) solution (ATCC). They were cultured in F-12K medium (ATCC) and incubated at 37 °C with 5% CO_2_ atmosphere in a Thermo Fisher Scientific (Rochester, NY, USA) incubator. Kaighn’s modification of Ham’s F-12 medium, containing 2 mM l-glutamine, 1500 mg/L sodium bicarbonate, 10% fetal bovine serum, and 1% penicillin-streptomycin (100 U/mL penicillin and 100 ug/mL streptomycin), was used.

Passage number eight was used for all experiments. After the targeted cell concentrations were achieved, the prostate cancer cells were deposited on the patterned silicon substrates in BioLite 35 mm tissue culture dishes. Three batches of cells with incubation periods of 30 min, 24 h, and 72 h were performed. The cell culture medium was subsequently removed and the silicon chips with adhered cells were washed twice with an equal volume of PBS (Thermo Scientific BupH phosphate-buffered saline, Waltham, MA, USA). Unless otherwise stated, the concentration used for these experiments was approximately 105 cells/mL. Cell concentrations were determined by using a hemocytometer (Hausser Scientific Co., Horsham, PA, USA) with cells stained with Trypan Blue (Lonza Walkersville Inc., BioWhittaker, Walkersville, MD, USA).

Cell fixing was conducted at room temperature by submerging chips in 4% paraformaldehyde solution for one hour. After washing the chips with PBS, they were kept submerged in PBS at 4 °C until staining. This is a well-established fixation process developed for the nuclear morphology studies in the literature [8,22,23,33,34]. Fixed PC3 cells were fluorescently stained for F-actin (phalloidin, CytoPainter F-actin Staining Kit, abcam^®^, Cambridge, MA, USA) and nuclei (4′,6-diamidino-2-phenylindole, DAPI, Life Technologies, Carlsbad, CA, USA). First, red fluorescent phalloidin conjugate was prepared according to the manufacturer's specified protocol. Cells were then rinsed twice with PBS prior to permeabilization with 0.1% Triton X-100 (Sigma-Aldrich Corporation, St. Louis, MO, USA) in PBS for five minutes at room temperature. After permeabilization, cells were rinsed twice with PBS and incubated in the dark with red fluorescent phalloidin conjugate at room temperature for 45 min. After incubation with phalloidin, the chips were rinsed two more times with PBS. DAPI counterstain was prepared according to the manufacturer’s specified protocol. Following F-actin staining, PC3 cells were incubated in the dark with 300 nM DAPI in PBS for three minutes at room temperature. Chips were then rinsed twice in PBS and remained submerged in PBS until imaging. Optical inspections were conducted with a Leica SP5 laser scanning confocal microscope (Leica Microsystems GmbH, Wetzlar, Germany) at the University of Guelph. The specimens were submerged in room temperature PBS during the imaging process. This instrument can acquire multiple channels of fluorescent signals simultaneously.

## 3. Results and Discussion

### 3.1. Silicon Pillars

**Figure 2:**
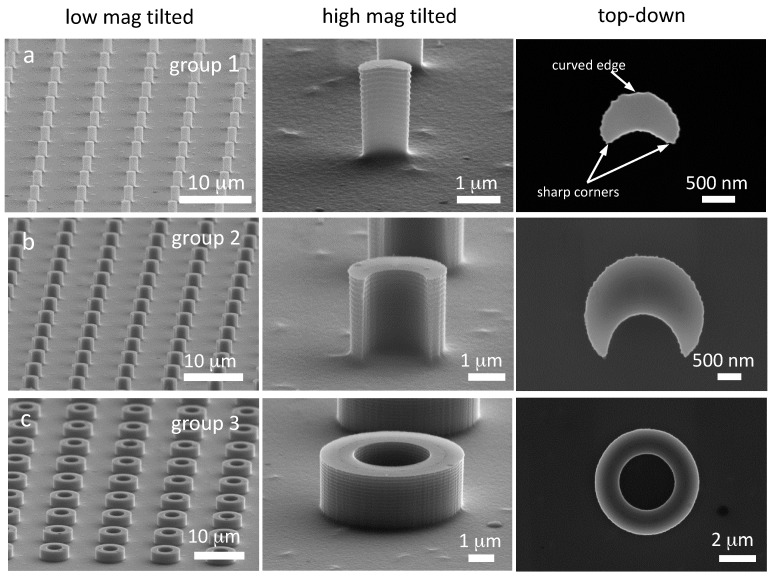
SEM micrographs of silicon pillars with complex cross-sectional geometries examined in this work: (**a**) group 1, small C-shaped pillars; (**b**) group 2, large C-shaped pillars; and (**c**) group 3, hollow pillars.

Representative scanning electron micrographs of silicon pillars with three different cross-sectional geometries are displayed in Figure 2a–c. They were fabricated on the same silicon wafers simultaneously and have an identical height of ~2.2 μm. C-shaped pillars with outer diameters of ~1.2 and ~2.7 μm are shown in Figure 2a,b. Each C-shaped pillar consists of two sharp corners and curved edges as labeled in Figure 2. Hollow-shaped columnar pillars were also examined and their micrographs are shown in Figure 2c. The pillars were positioned in an orthogonal orientation. Physical dimensions of these structures are listed in Table 1. Each of these pillar arrays covers a square area of ~3.5 mm × ~3.5 mm. The pillar's center-to-center distances of the three specimen groups—namely 1, 2, and 3—are approximately 6.2, 7.6 and 10.5 μm, respectively.

More importantly, the gaps between adjacent pillars—regions that allow portions of cells to extend into during the spreading process—are identical for all pillars (~5 μm). Badique et al. [24] and Wang et al. [8] have suggested that pillar spacing is an important parameter for nuclear deformation. Maintaining identical gap spacing between the pillars will reduce uncertainties of flow dynamic variations among pillars with different cross-sectional geometries and allow for a direct comparison of results from these three pillar groups. Unlike cylindrical pillars with axisymmetric geometry, the C-shaped pillars fabricated in this work provide a unique surface topography that produces non-axisymmetric mechanical stress states on the attached cells. Measured from the top-down views, the tip radii at the corners of the small and large C-shaped pillars are approximately 80 nm and 136 nm, respectively. Since the mechanical stress concentration factor increases with reduced tip radius [28], the smaller C-shaped pillars with sharper corners and edges are expected to induce greater stress on the cells. In contrast, the 5.6 μm outer diameter hollow pillars are axisymmetric structures with low stress concentration and are provided as baselines for comparison with the C-shaped pillars.

The precise contact pressure profiles produced by these three pillar structures on the adherent cells are difficult to determine because they require detailed information on the pillar-cells’ three-dimensional contact profile, the mechanical properties of the live cells, and the directions and magnitudes of applied forces—which are hard to define as they change during the dynamic cell-spreading process. However, by using Equations (1)–(3) we can describe the effects of tip radius on contact pressure under simple loading geometries, such as spherical and cylindrical contacts on an elastic half-space. By reducing the spherical tip radius from 5.6 μm to 136 nm and 80 nm, the maximum contact pressure increases ~10.9 and ~16.0 times, respectively. The maximum contact pressures for smaller cylindrical contacts are ~5.4 and ~7.4 times greater, respectively. In both contact cases, the maximum pressure increases with the reduced radius of the structure. As mentioned above, it is important to note that the actual stress profiles experienced by the adherent cells can be more complex than the two simple models evaluated here; however, they do serve as a demonstration of the pillar corner and edge radius effects.

### 3.2. Cells on Bare Silicon Substrates

**Figure 3:**
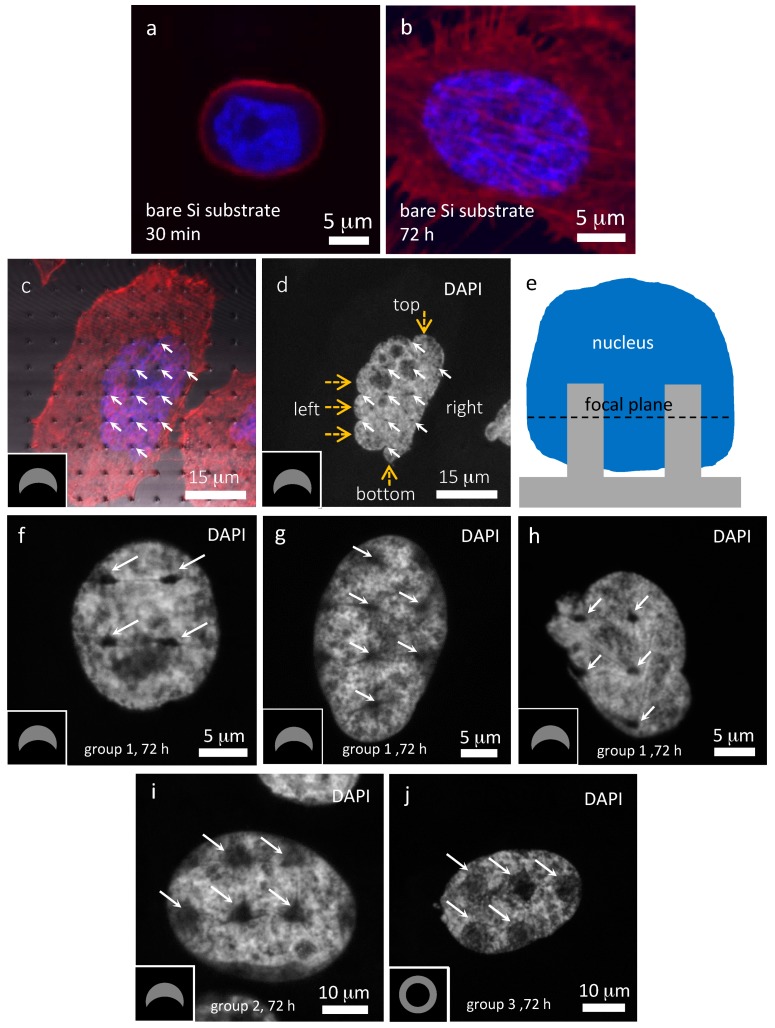
Confocal fluorescence micrographs of PC3 cells incubated for (**a**) 30 min and (**b**) 72 h on bare Si substrate; (**c**) Composite and (**d**) DAPI-only micrographs of PC3 cells incubated for 72 h on small C-shaped pillars (group 1). Pillar locations are indicated by solid arrows while bulged edges of the DAPI-stained materials are highlighted by dashed arrows in (**c**). A schematic drawing of the focal plane used in these micrographs is shown in (**e**). Representative micrographs of PC3 cells incubated for 72 h reveal fine line structures emanating from the small C-shaped pillars are displayed in (**f**–**h**). Lines in (**f**) are connected with the adjacent pillars while lines in (**g**) and (**h**) are not. Confocal micrographs of 72 h incubated PC3 cells on large C-shaped and hollow-shaped pillars are displayed in (**i**,**j**), respectively. Inset drawings indicate the pillar orientations.

A typical top-down high-resolution confocal micrograph of a PC3 cell incubated for approximately 30 min on a flat, smooth bare silicon substrate without pillar patterns is shown in Figure 3a. This micrograph shows that the cell is compact with near circular geometry and the nucleus is located at approximately the center of the cell. This is expected as this cell has only been deposited on the rigid surface for a short period of time. Additionally, cytoskeletal protein F-actin projection was observed only at the cell periphery where the cells contact the substrate. The average diameter of ten randomly-selected 30-min incubated cells was 16.6 ± 2.9 μm. Their sizes are statistically indistinguishable from results published in the literature of 15.1 ± 2.6 μm [35] and reported by Nexcelom Bioscience LLC of 18.08 ± 2.69 μm [36]. This demonstrates that the cell morphology was not grossly altered by the fixation processes. Representative confocal inspections were also conducted on PC3 cells adhered to flat, smooth, bare silicon substrates after approximately 72 h of incubation; these are shown in Figure 3b.

This DAPI and fluorescent phalloidin-stained composite micrograph shows that the cell has spread and successfully attached to the flat, smooth, bare silicon substrate. This cell contains a large number of F-actin-rich filopodia and lamellipodia. Some areas within the nucleus, as shown in Figure 3a,b, were not stained as intensely by DAPI and appear dim or dark. These regions are randomly distributed with varying sizes and shapes, and most likely correspond to different nuclear components, such as nucleoli or euchromatin compartments.

### 3.3. Cells on Patterned Silicon Substrates

Representative confocal micrographs of PC3 cells incubated on small (group 1) and large C-shaped (group 2) pillars for 72 h are shown in Figure 3. An example of PC3 cells incubated on small C-shaped pillars (group 1) for 72 h is displayed in Figure 3c,d. These micrographs were collected at a focal plane below the top of the pillar, as schematically illustrated in Figure 3e. A composite micrograph of blue DAPI, red phalloidin, and gray-scale optical reflection images of the silicon-patterned structures is displayed in Figure 3c. The inset schematic drawing indicates the orientation of the small C-shaped pillars on the substrate. Pillar locations are highlighted with solid arrows unless otherwise stated. This micrograph shows many filopodia and lamellipodia are observed on this cell, with the majority of these being attached to the substrate. It is interesting to note that some appear preferentially bonded to the pillars. Such selective attachment behaviors of actin structures on small pillars have been previously reported by Albuschies and Vogel [37] with human dermal foreskin fibroblast cells. In addition, Jahed et al. [38] showed 3T3 Swiss Albino fibroblasts cells preferentially sensing and remaining attached to metallic nanopillars. Figure 3d shows the DAPI-stained areas of this cell in gray-scale. Detailed inspections of this image reveal the locations of C-shaped pillars as small solid dark spots (highlighted with white solid arrows). This micrograph also showed other features with a varying intensity of DAPI fluorescence which may represent the locations of different sub-nuclear organelles. At least eleven silicon pillars can be identified surrounded by the nucleus displayed in Figure 3d with six located at the center of the stained region, while the rest are positioned along the edges. Figure 3d shows this nucleus to have distinctly bulged edges (highlighted with yellow dashed arrows) at the top, bottom, and left portions of the nucleus. They are likely created by the flow of membrane-bound nuclear material in between the stiff silicon pillars during the cell spreading process. The nucleus and its membrane are expected to experience highly-localized mechanical stress at the pillar locations where the material flows are restricted and the nuclear membrane is severely deformed in order to conform to the shape of the silicon pillars.

Another important characteristic feature observed in some of the cells deposited on the small C-shaped pillars (group 1) are distinct fine dark line structures revealed in the DAPI-labeled micrographs collected at focal planes below the pillar top, as schematically illustrated in Figure 3e. Figure 3f shows examples of such fine dark lines in a DAPI-stained area radiating outward from the pillar locations and the inset drawing indicates the orientation of the small C-shaped pillars on the substrate. The locations of four pillars are highlighted with solid arrows. These dark lines, less than a micron wide, can be clearly seen emanating from all four pillars located near the center of the nucleus, as displayed in Figure 3f. They are uniquely different from the randomly-distributed and irregularly-shaped dim background features produced by other sub-nuclear organelles. The lack of DAPI fluorescence signal in these fine line structures indicates a low concentration of nuclear DNA in these features. Some nuclei, as shown in Figure 3g,h, contain line features that are not connected to adjacent pillars and have lengths in the micron scale. Additional micrographs are revealed in Supplementary Figure S1 to demonstrate the reproducibility of these dark line features. The DAPI-labeled micrographs shown in Figure 3f–h also reveal that the pillar's dark spots resembling filled semi-circles rather than their true C-shaped geometry. This indicates that the pillars are not in direct contact with the DNA. A possible reason is that the nuclear envelope and the plasma membrane remain intact at the cell-pillar interface and provide a gap between the DNA and the pillar. These pillars are unable to rupture the cell membranes and make direct contact with DNA materials. Instead, cells putatively engulf and wrap around the pillars. This is expected as several previous studies [39,40,41,42] have shown that micropillars are unlikely to penetrate the cell membranes due to their large dimensions and small height to diameter aspect ratios. The narrow dark spacing between the pillar and the DAPI stained DNA shown in Figure 3f,h may be filled with other cellular components, such as the plasma membrane, cytoplasmic components, the nuclear envelope, or other sub-nuclear organelles. Hence, the dark spots do not resemble pillar true geometries.

**Figure 4:**
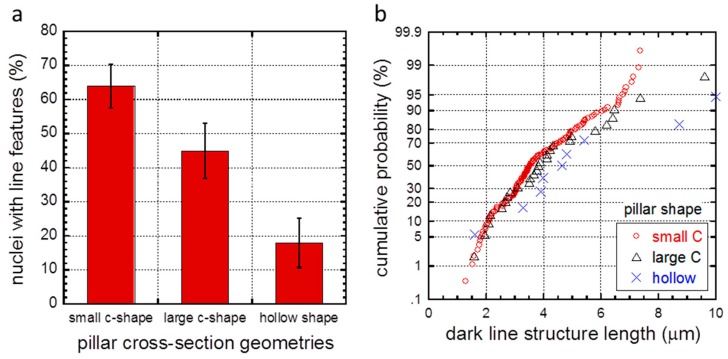
(**a**) Population of cell nuclei with slit features for three different pillar cross-section geometries. Slit length distributions on various shaped pillars are displayed in (**b**).

Random inspection of 55 cells deposited on small C-shaped pillars (group 1) revealed that 35 cells (64 ± 6%) exhibited these dark line structures where the data spread corresponds to one standard error. These results are plotted in Figure 4a. The length distributions of 132 dark lines surrounding small C-shaped silicon pillars were measured and displayed in Figure 4b. This cumulative probability plot shows the line lengths are in the range of ~1.3 and ~7.3 μm, with the average value of 3.8 ± 1.4 μm. The data spread represents one standard deviation. On average there are ~3.8 lines formed in each cell that contains dark lines. Among the 132 lines inspected, 104 of them (79% of the line population) extended from the sharp corners, while 28 lines (21%) emanated from the curved edges. This demonstrates that the sharp corners of the C-shaped pillars are the more likely locations to form dark line structures.

Careful inspection of PC3 cells incubated for approximately 72 h on large C-shaped pillars (group 2) revealed similar sub-micron-scale dark line structures, as shown in Figure 3i. The large C-shaped pillars surrounded by the nucleus are labeled with arrows and the separation distance between the pillars is identical to the small C-shaped pillars (~5 μm). This confocal micrograph shows that the dark spots where the pillars are located do not bear a resemblance to a true pillar C-shape but instead have filled-triangular profiles. Interestingly, several of the fine dark line features extend from the tips of these triangles—an indication that they may be structurally connected. Random inspections of 38 PC3 cells attached on large C-shaped pillars (group 2) showed that 17 of them (45 ± 8%) exhibit line features as shown in Figure 4a. These results show that fewer PC3 cell nuclei (45% vs. 64%) exhibit line structures when they are deposited on the large C-shaped pillars in comparison to the smaller counterparts.

The length distributions of 27 dark line structures surrounding the large C-shaped pillars are plotted in Figure 4b. The average dark line length in these cells is 4.2 ± 1.9 μm where the data spread corresponds to one standard deviation. These results are statistically indistinguishable from the small C-shaped pillars results. The average number of dark lines observed in cells contacting large C-shaped pillars is ~1.6—two times fewer than those observed in small C-shaped pillars of 3.8. One primary reason is a reduced number of dark lines originated from the curved edges of the large C-shaped pillars. Inspection of cells on large C-shaped pillars show 24 out of 27 lines (89%) originated from the two sharp corners of pillars but only three lines (11%) were extended from the curved edges. The lack of dark lines emanating from large C-shaped pillar curved edges may suggest that their formation may be related to the dimension of the pillars. Large C-shaped pillar diameters are more than two times larger than the small C-shaped counterpart (1.2 vs. 2.7 μm). 

Results shown in Figure 3 and Figure 4 indicate that the dark line formation process depends on the pillar’s cross-sectional geometry and may be influenced by the sharp corners and curved edges of the small C-shaped pillars. These sharp features are expected to magnify the mechanical forces produced by the cytoskeletal structures pushing downward on the nuclei [4,28]; hence, greater indentation forces are applied to the cells. To test this hypothesis, cells were deposited on hollow-shaped pillars fabricated on the same substrate as the C-shaped pillars. The separation distance between the hollow pillars is identical to the C-shaped structures of ~5 μm in order to reduce the uncertainties related to dynamic flow of material between the pillars. Since these hollow pillars have axisymmetric geometry with smooth, curved surfaces, the amount of induced stress is expected to be low when compared to C-shaped pillars with sharp corners.

A representative image of a PC3 cell that was incubated on hollow pillars for approximately 72 h is shown in Figure 3j and does not show distinct line structures. Random sampling of 28 cells indicated that only five nuclei (18 ± 7%) showed dark line structures, as shown in Figure 4a. These results signify that the important factor for the dark line formation is pillar’s cross-sectional geometry. The line length distributions on these cells are shown in Figure 4b and show the dark lines observed on hollow-shaped pillars have average lengths of 5.1 ± 2.6 μm. While the average length is longer than those observed in C-shaped pillars, the differences are statistically insignificant due to the large data standard deviation. Furthermore, the average number of lines observed surrounding each cell is 1.8 which is approximately the same as those on the large C-shaped specimen. Experiments were repeated with cells incubated for approximately 24 h and showed similar dark line structures. Examples of 24 h cell incubation on small C-shaped pillars (group 1) and large C-shaped pillars (group 2) are shown in Supplementary Figure S2a,b respectively.

**Figure 5:**
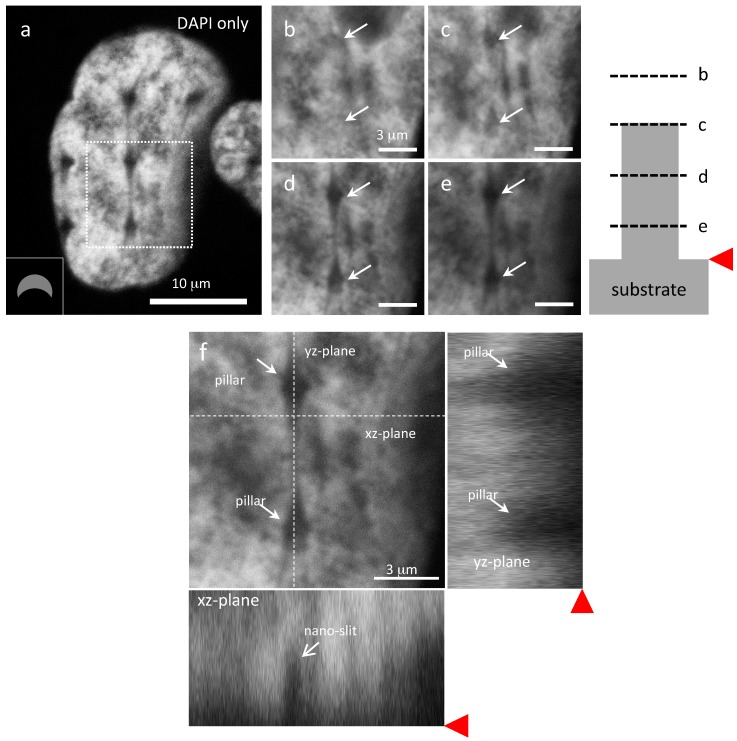
(**a**) DAPI only micrograph revealing fine line structures in cells incubated on small C-shaped pillars. High magnification images of a representative nucleus surrounding two small C-shaped pillars imaged in various focal planes are shown in (**b**–**e**). The corresponding focal plane locations are illustrated in the schematic drawing. Orthogonal views of this nucleus are shown in (**f**) revealing two pillars and a nano-slit. Red triangle pointers indicate substrate surface locations.

To understand the three-dimensional configurations of these line features, additional inspections of the PC3 cells were conducted at multiple focal planes. High-magnification DAPI-stained micrographs of the fine line structure from the large nucleus displayed in Figure 5a were collected and are shown in Figure 5b–e. The locations of these focal planes are schematically illustrated in the accompanied diagram. The z-section image sequence begins at a focal plane slightly above the top of small C-shaped pillars, and sequentially downward to the pillar base. White solid arrows in the micrographs indicate where the two pillars are located. No fine line structures or pillar dark spots are observed in the micrographs taken at focal planes above the pillar tops (Figure 5b). Faint impressions of two C-shaped pillars appear in Figure 5c with the focal plane located near the top of the pillars, but no dark line is observed in this micrograph. When the focal plane is located below the pillar top level, as the micrograph shown in Figure 5d, a faint fine dark line that connects the pillars is clearly visible. In addition, the two pillar dark spots displayed in Figure 5d show they do not resemble the true pillar C-shaped profile but instead a tear-drop shaped geometry where the tail portions of the feature narrows to form fine dark lines connecting adjacent pillars. This indicates that the pillar's dark spots and the fine dark lines are structurally connected.

Furthermore, the tear-drop shaped and fine dark line structures are observed at other focal planes further down toward the substrate (see Figure 5e). This suggests that these observed features are cross-sectional views of a thin continuous vertical slit connecting adjacent pillars. It begins below the top of the pillars and extends downward. The geometric structure of this nanometer scale slit is further confirmed by the orthogonal views of this cell nucleus as shown in Figure 5f where the cross-section locations are labeled with dashed lines. A nano-slit is clearly visible on the xz-plane.

**Figure 6:**
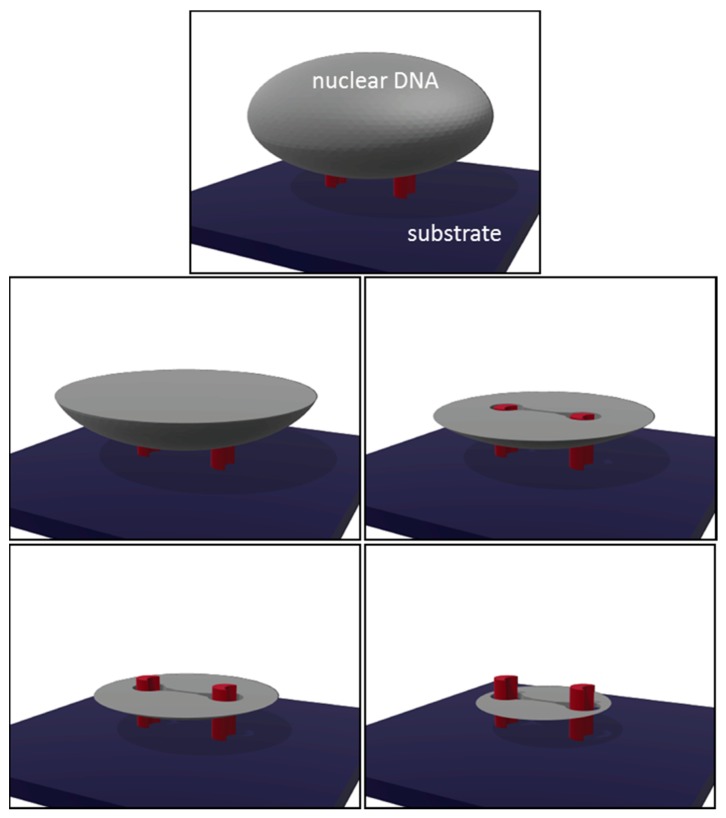
Schematic drawings of nuclear DNA elements (gray) surrounding two C-shaped pillars (red) at different cross-sectional planes above and below the pillars. Note the DNA materials are not in direct contact with the pillars. The tear drop-shaped openings in between the pillars indicate the slit location.

Schematic cross-sectional drawings of nuclear DNA elements surrounding two C-shaped pillars are shown in Figure 6. The drawings indicate that the DNA elements do not make direct contacts with the pillars. Instead, tear-drop shaped gaps are formed between the DNA and the pillars where they may be filled with the plasma membrane, nuclear envelope, or other sub-nuclear organelles that were not stained by DAPI. To the best of our knowledge, this is the first literature report of silicon micropillar-induced nano-slit structures within human prostate cancer cells. Confocal micrographs reveal that these continuous slits of materials are depleted of DNA. 

**Figure 7:**
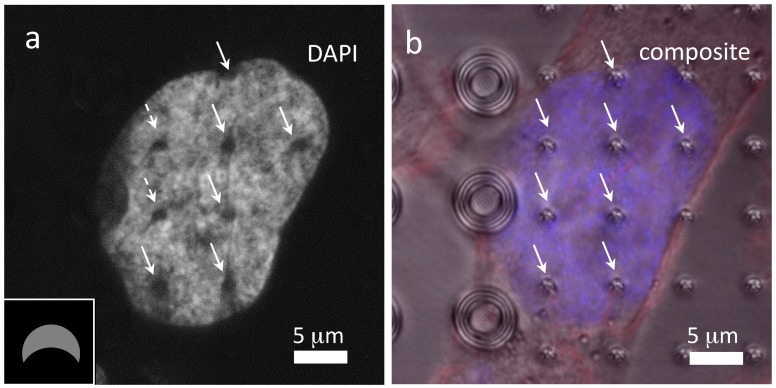
(**a**) DAPI only and (**b**) composite top-down micrographs of a cell adhered on small C-shaped and hollow pillars. The locations of the pillars are highlighted with arrows. Cells were incubated on the pattern substrate for 72 h. Inset drawing indicates the pillar orientation.

The formations of these nanometer-scale thick slits are not only observed in cells deposited on a uniform array of C-shaped pillars, but have also been confirmed in cells that are simultaneously contacting pillars with different cross-sectional geometries, as revealed in Figure 7a,b. These confocal fluorescence micrographs show the DAPI and composite images of a cell that is adhered concurrently to two different arrays of hollow silicon pillars (group 3) and small C-shaped (group 1) geometries. The majority of the cell nucleus is surrounding the small C-shaped pillars (highlighted with arrows) while the remaining part of the cell is extended to the hollow pillars. Even though the cell contacts drastically different surface topographies, the DAPI-strained DNA materials still show slits as those shown above. It is remarkable that slits can be observed in the two C-shaped pillars that are less than 5 μm away from the hollow pillar arrays (marked with dash arrows). However, it is unclear if the orientations and the lengths of these slits have been influenced by the nearby hollow pillar arrays. The evidence presented here indicates that the slit formation mechanism may be driven by a localized effect at the micron-scale that is determined by the pillar geometries.

The confocal micrographs presented above show that sub-micron-scale stiff silicon pillar structures with C-shaped geometries produce nanometer-scale slit features in the PC3 cells. Results also revealed that not only can the nuclei be reshaped, but they may also be sensitive to the cross-sectional geometries of the surface topography. The exact origin and mechanisms of these slit formations have yet to be identified. One possible explanation may be related to the pillar/cell contact geometries. As cells spread on textured silicon surfaces their cytoskeletons push the nuclei and other sub-cellular organelles downward and press against the pillars [22,23]. This resembles nano-indentations of viscoelastic materials (cytoplasm and nuclei) contained within elastic membranes (plasma membranes and nuclear membranes) by a series of complex-shaped silicon cylinders. Hanson et al. showed that nuclear deformation on nanopillars is determined by nuclear stiffness as well as cytoskeletal forces, and that the geometry of nanopillar arrays highly influences nuclear deformation. As live cells press against the silicon pillars, the plasma membrane, cytoplasm, and nuclear envelope deform and occupy the space in between the pillars. For asymmetric hollow pillars, deformations near the contact rims were evenly distributed. However, membrane deformations on complex-shaped pillars, such as C-shaped structures, were more severe near the two sharp corners. This may cause the plasma membrane and nuclear envelope to fold inward and form creases near the corners. Since the membranes do not contain DNA, the creases formed resemble thin dark lines or slits, as illustrated in Figure 3 and Figure 5.

This work demonstrates that PC3 cell mechanical contact responses to stiff silicon micro-pillars are more complex than previously understood and open a new direction of investigation in this field. Future research should include in situ live cell time-lapse microscopy imaging studies of these cancer cells with different pillar geometries. This focus could gain temporal information about how the slits are formed and help develop an understanding of whether the presence of the nano-slits will prevent or restrict the transport of the nuclear material between different parts of the nucleus. The effects of these structures on basic cell biological functions, such as proliferation, metabolism, mitosis, and cell division, should be investigated and compared between cancer and normal cells. During typical cell division processes, DNA molecules are replicated and chromatin molecules are being condensed into chromosomes (prophase). Eventually, the chromosomes or sister chromatids are being pulled by the microtubules to the opposite ends of the cells (metaphase and anaphase). However, it is unclear how the slits, which may act as physical barriers, affect the chromosome replication processes and microtubule motions. Even if cells were successfully divided, it is unclear if the presence of slits will induce chromosome replication errors. To address these questions, further experiments are needed to compare the DNA sequences among cells grown on the patterned surfaces with different passages. Other essential macro molecules, such as ribonucleic acids (RNAs) and peptide chains, should also be analyzed from different passages to understand if the cell functions are compromised.

Additional investigation is also required to determine how the response of PC3 cells to silicon micropillars compares with other cancer and healthy cell types. One type of the potentially healthy cells to be investigated are human fibroblast cells. Their average nuclear dimensions are similar to the PC3 cells, on the order of tens of microns, when they adhered and spread on the patterned silicon surfaces. This type of study will provide a comparison of surface topographic responses on normal and cancerous cells. Investigations of slit formations should also be conducted on other immortalized cancerous cell lines, such as HeLa cervical cancer cells, to determine if the slit formation is a mechanical contact phenomenon unique to cancerous cells or PC3 cells. Finally, other pillar cross-sectional geometries with higher stress concentration points should also be investigated to understand the effect of introducing a stress field to sub-nuclear organelles.

## 4. Conclusions

The response of human prostate cancer cell line PC3 nuclei on nanometer-scale textured silicon micropillars with complex cross-sectional geometries was studied using laser scanning confocal fluorescence microscopy. Results show the formation of nanometer scale slit structures in the DAPI-stained elements near some pillars where the number of slits formed is related to pillar size and cross-sectional morphology. These features develop most frequently in small C-shaped pillars, followed by large C-shaped pillars and hollow circular pillars. DAPI fluorescence micrographs reveal a low concentration of DNA in these slit structures, which are only observed in the portion of the nucleus located below the pillar top surfaces.

## Figures and Tables

**Table 1:** Physical dimensions of micron and sub-micron scale silicon pillars. Statistical distributions of PC3 cells that show fine line features after incubation on silicon substrates with various pillar geometries for approximately 72 h. Data spreads correspond to one standard error.

Group	Pillar Shape	Outer Diameter (μm)	Wall Thickness (μm)	Center-to-Center Distance (μm)	Nuclei Inspected	Nuclei with Line Features	% Nuclei with Line Features
1	small C-shape	1.2	--	6.2	55	35	64 ± 6
2	large C-shape	2.7	--	7.6	38	17	45 ± 8
3	hollow	5.6	1.3	10.5	28	5	18 ± 7

## Supplementary Materials

The following are available online at www.mdpi.com/1996-1944/10/8/892/s1. Figure S1: (a) show a DAPI only micrograph with dark line structures radiating out at the corners of the small c-shaped pillars (group 1). Composite image of the same cell is shown in (b). This cell was incubated on patterned silicon substrate for 72 h. Inset indicates the pillar orientation, Figure S2: Representative DAPI only and composite confocal micrographs of dark line structures radiating out at the corners of (a) small C-shaped pillars (group 1) and (b) large C-shaped pillars (group 2) respectively. Cells were incubated on patterned substrate for 24 h. Insets indicate the orientation of the pillars.
