# Supplementary material for: Mechanical Contact Characteristics of PC3 Human Prostate Cancer Cells on Complex-Shaped Silicon Micropillars
Source: Materials (Basel). 2017 Aug 2;10(8):892. doi: 10.3390/ma10080892 (PMC5578258; doi:10.3390/ma10080892)
Supplement: Supplementary file 1 [file materials-10-00892-s001.pdf]

Supplementary Materials: Mechanical contact characteristics of PC3 human prostate cancer cell on complex shaped silicon micropillars

Brandon B. Seo [1], Zeinab Jahed [2], Jennifer A. Coggan [3], Yeung Yeung Chau [3], Jacob L. Rogowski [1], Frank X. Gu [1], Weijia Wen [4], Mohammad R. K. Mofrad [2] and Ting Y. Tsui [1,*]1. Heading

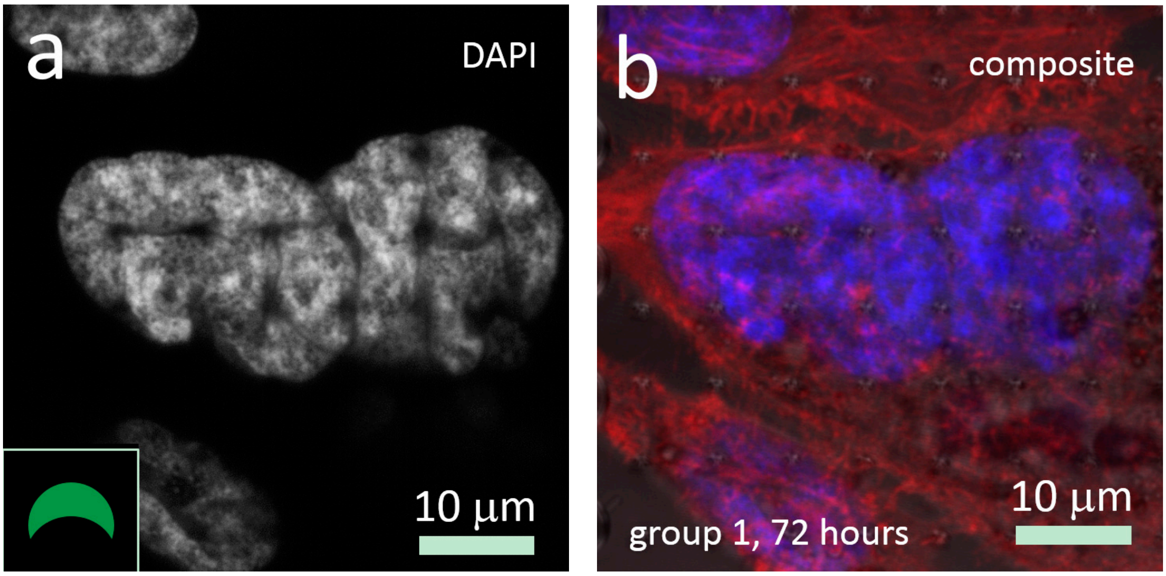

Figure S1. (**a**) show a DAPI only micrograph with dark line structures radiating out at the corners of the small c-shaped pillars (group 1). Composite image of the same cell is shown in (**b**). This cell was incubated on patterned silicon substrate for 72 hours. Inset indicates the pillar orientation.

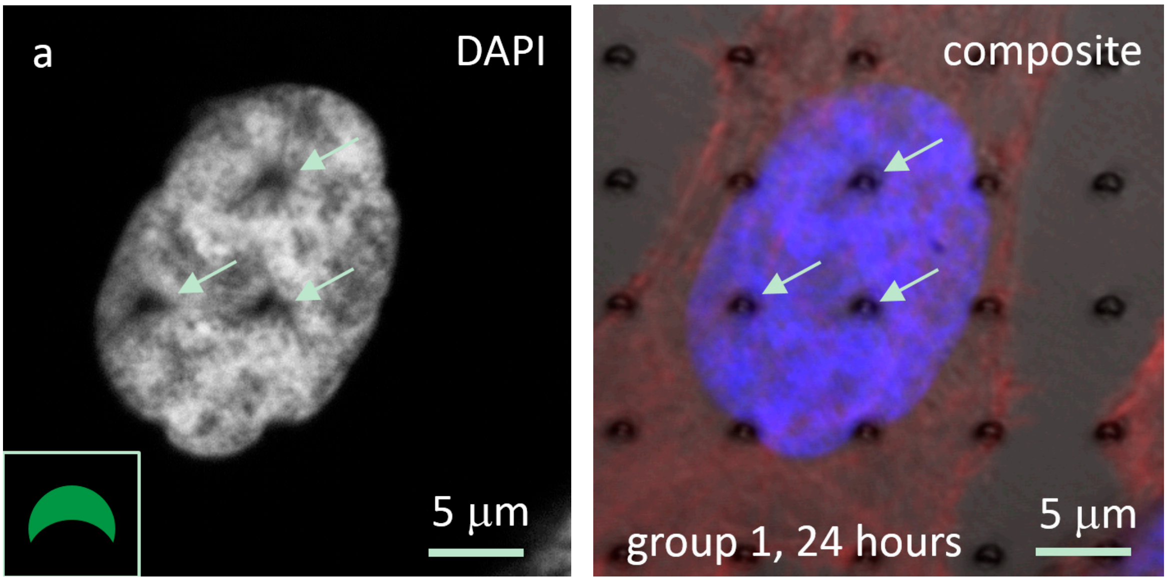

Figure S2. *Cont.*

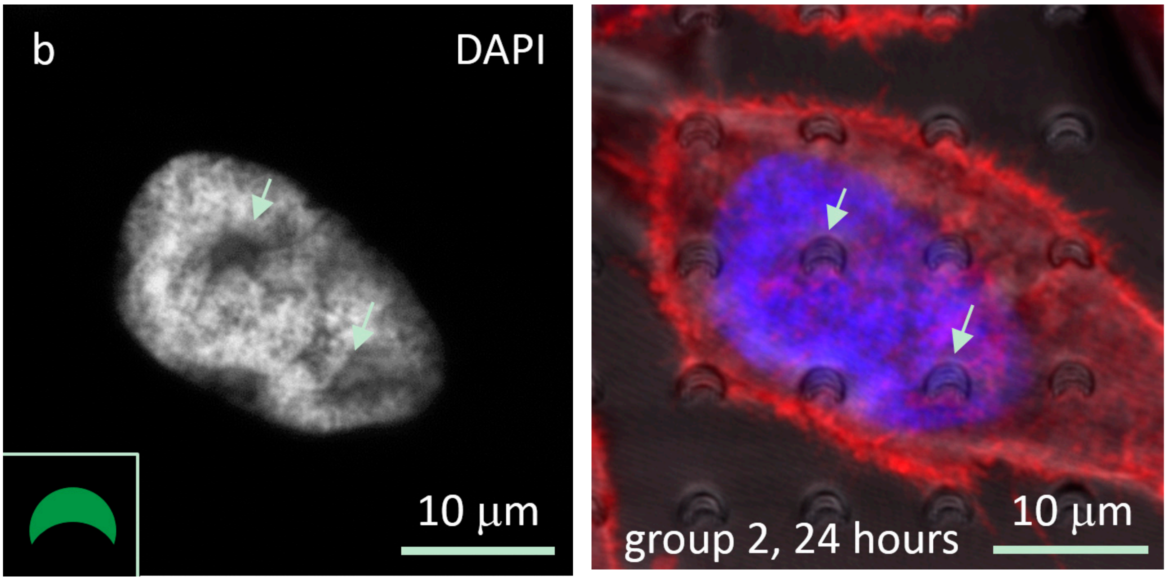

Figure S2. Representative DAPI only and composite confocal micrographs of dark line structures radiating out at the corners of (**a**) small C-shaped pillars (group 1) and (**b**) large C-shaped pillars (group 2) respectively. Cells were incubated on patterned substrate for 24 hours. Insets indicate the orientation of the pillars.
